# Community-Associated Methicillin-Resistant *Staphylococcus aureus*, Iowa, USA

**DOI:** 10.3201/eid1510.080877

**Published:** 2009-10

**Authors:** Philip Van De Griend, Loreen A. Herwaldt, Bret Alvis, Mary DeMartino, Kristopher Heilmann, Gary Doern, Patricia Winokur, Diana DeSalvo Vonstein, Daniel Diekema

**Affiliations:** The University of Iowa Carver College of Medicine, Iowa City, Iowa, USA (P. Van De Griend, L.A. Herwaldt, B. Alvis, K. Heilmann, G. Doern, P. Winokur, D. Diekema); The University of Iowa Hygienic Lab, Iowa City (M. DeMartino); The University of Iowa College of Public Health, Iowa City (L.A. Herwaldt); The University of Iowa Hospitals and Clinics, Iowa City (L.a. Herwaldt, D. Diekema); The Iowa City Veterans Administration Medical Center, Iowa City (P. Winokur, D.D. Vonstein, D. Diekema)

**Keywords:** Methicillin-resistant Staphylococcus aureus infections, MRSA, community-associated MRSA, surveillance, bloodstream infection, Iowa, bacteria, Staphylococci, research

## Abstract

We performed antimicrobial drug susceptibility testing and molecular typing on invasive methicillin-resistant *Staphylococcus aureus* (MRSA) isolates (n = 1,666) submitted to the University of Iowa Hygienic Laboratory during 1999–2006 as part of a statewide surveillance system. All USA300 and USA400 isolates were resistant to <3 non–β-lactam antimicrobial drug classes. The proportion of MRSA isolates from invasive infections that were either USA300 or USA400 increased significantly from 1999–2005 through 2006 (p<0.0001). During 2006, the incidence of invasive community-associated (CA)–MRSA infections was highest in the summer (p = 0.0004). Age <69 years was associated with an increased risk for invasive CA-MRSA infection (odds ratio [OR] 5.1, 95% confidence interval [CI] 2.06–12.64), and hospital exposure was associated with decreased risk (OR 0.07, 95% CI 0.01–0.51).

## Abstract 2

The proportion of invasive methicillin-resistant *Staphylococcus aureus* infections caused by USA300 increased in 2006.

Methicillin-resistant *Staphylococcus aureus* (MRSA) emerged in the 1960s and has since become a major cause of illness and death in the healthcare setting (*1**,**2*). Risk factors for infection with healthcare-associated MRSA (HA-MRSA) include hospitalization, residence in a long-term care facility, older age, invasive devices (e.g., catheters, feeding tubes), and exposure to antimicrobial agents. HA-MRSA isolates are often resistant to several antimicrobial drug classes in addition to β-lactams (*3*).

In the 1990s, investigators began describing serious MRSA infections among persons who did not have typical risk factors for infections with this organism (*2**,**4**–**8*). These community-associated MRSA (CA-MRSA) infections affected young, healthy persons (*4**,**5**,**7*) and were associated with factors such as participating in contact sports, sharing towels or athletic equipment, using illegal intravenous drugs, and living in crowded or unsanitary areas (e.g., prisons, hurricane evacuee centers) (*9**,**10*).

Pulsed-field gel electrophoresis (PFGE) demonstrated that MRSA strains causing these community-associated infections (USA300 and USA400) were different than those causing healthcare-associated infections (USA100 and USA200) (*11*). USA300 and USA400 MRSA strains typically have the staphylococcal cassette chromosome (SCC) *mec* type IV, not the SCC*mec* type II carried by most USA100 and USA200 isolates (*12*). In addition, USA300/400 isolates usually carry the gene that encodes the Panton-Valentine leukocidin (pvl), a bicomponent (*luk*F-PV and *luk*S-PV) pore-forming leukotoxin (*8**,**13**–**15*). Currently, the role of PVL in the pathogenesis of infections caused by USA300/400 isolates is controversial. Epidemiologic studies and a study by **Labandeira-Rey** et al. suggest that PVL is associated with virulence and causes the necrosis characteristic of infections with these strains (*16*). In contrast, a study by Voyich et al. found no difference in virulence between the wild-type parent strains and the isogenic knockout strains that did not produce PVL (*17*).

A recent multicenter study by Moran et al. showed that USA300 MRSA is now the most common cause of skin and soft tissue infections (SSTIs) among adults seeking treatment in emergency departments in 11 large metropolitan areas (*15*). USA300 also causes serious invasive infections such as necrotizing pneumonia, bloodstream infections, and surgical site infections, some of which are acquired in hospitals (*18**–**22*). Although most USA300 and USA400 isolates are currently resistant to fewer classes of antimicrobial drugs than are HA-MRSA isolates (*13*), a recent paper by Han et al. identified a USA300 subtype that is resistant to erythromycin, clindamycin (constitutive), tetracycline, mupirocin, and fluoroquinolones (*23*).

Most epidemiologic studies of CA-MRSA have examined isolates from SSTIs infections (*7**,**15**,**18*), and most studies that evaluated patients with invasive disease have involved single healthcare facilities (*21**,**24*) or isolates obtained primarily from large urban areas (*22*). We describe the molecular epidemiology of invasive infections caused by USA300 and USA400 in a rural state. We characterized invasive MRSA from 1999–2005 (select isolates) and in 2006 (all isolates) submitted to the statewide surveillance system in Iowa for invasive MRSA infections.

## Methods

As part of a statewide surveillance system, the Iowa Department of Public Health has mandated since 1999 that clinical microbiology laboratories submit invasive isolates of MRSA to the University Hygienic Laboratory (UHL), Iowa’s public health laboratory (*25**,**26*). After performing antimicrobial drug susceptibility testing on all isolates, we further characterized (by PFGE, PVL detection, and SCC*mec* typing) all isolates from 1999–2005 that were resistant to <3 non–β-lactam antimicrobial drug classes (i.e., consistent with USA300/400) and all 343 isolates from unique patients with invasive infections submitted to the UHL during 2006.

### Antimicrobial Drug Susceptibility Testing

All invasive MRSA isolates during 1999–2006 were tested for antimicrobial drug susceptibility by the broth dilution method described by the Clinical and Laboratory Standards Institute (*27*). Invasive isolates were defined as any organism from a normally sterile body site such as blood, cerebrospinal fluid, pleural fluid, joint fluid, or fluid from a liver abscess. Isolates from urine were excluded.

Isolates were tested for susceptibility to tetracycline, erythromycin, clindamycin, trimethoprim/sulfamethoxazole, gentamicin, levofloxacin, moxifloxacin, linezolid, daptomycin, vancomycin, and rifampin. Multidrug-resistant isolates were defined as MRSA isolates that were resistant to more than 3 of 8 representative antimicrobial drug classes: macrolides (erythromycin), lincosamides (clindamycin), quinolones (levofloxacin or moxifloxacin), tetracyclines, sulfa drugs (trimethoprim/sulfamethoxazole), aminoglycosides (gentamicin), glycopeptides (vancomycin), and rifampin.

### Molecular Typing and PCR to Assess SCC*mec* Type and Presence of the PVL Gene

PFGE was performed as previously described (*28*). Each gel accommodated bacteriophage Lambda ladders (at 3 places on the gel), DNA from 17 isolates, type strains for USA300 and USA400 from the Centers for Disease Control and Prevention (Atlanta, GA, USA), and *S. aureus* NCTC-8325 (at 3 places on the gel). The gel images were saved as TIFF files and BioNumerics computer software (Biosystematica, Llandysul, Wales, UK) was used to perform cluster analysis. Isolates were classified as the same strain if cluster analysis indicated that they were >80% similar. PFGE patterns for clinical isolates were compared visually and by computer-assisted gel analysis with the type strains for USA300 and USA400. We defined CA-MRSA as MRSA isolates in either the USA300 or the USA400 pulsetypes. Multiplex PCR was performed, as previously described, to type the SCC*mec* A (*29*) and to detect the PVL genes (*30*).

### Epidemiologic Data Collection

Epidemiologic data on the isolates were obtained from UHL. These data were age, sex, race/ethnicity, inpatient status, intensive care unit status, long-term-care facility status, hospital admission date, specimen type, specimen collection date, the hospital code number, and the Iowa Reporting Region. Isolates were considered to have been acquired nosocomially if the specimen culture date minus the admission date was >2 days.

### Statistical Methods

PFGE patterns and antimicrobial drug susceptibility test results were merged with the demographic data. These data were analyzed with SAS version 9.1 (SAS Institute, Cary, NC, USA) to assess trends in the frequency of USA300/400 in Iowa and to identify possible risk factors for invasive infections with these strains. We used χ^2^ and adjusted χ^2^ tests to analyze categorical data and linear regression and logistic regression to analyze continuous data. Alpha was set at 0.05 and all reported p values were 2-tailed.

Seasonality of infections was analyzed by χ^2^ analysis. Winter was defined as December 22 to March 19, spring as March 20 to June 20, summer as June 21 to September 22, and fall as September 23 to December 21.

The relationships between CA-MRSA and potential risk factors were assessed by univariate analysis. Subsequently, stepwise logistic regression was used to identify factors independently associated with invasive CA-MRSA infection.

## Results

Patients infected by USA300/400 MRSA were younger than those infected by other strains (p<0.0001 for both time periods; Tables 1, 2). During 2006, more males than females were infected with USA300/400 (p = 0.06). Most isolates during both time periods were obtained from blood cultures and the distribution of strains did not vary by body site. Most patients were hospitalized for their infections and the proportion of patients admitted to intensive care units did not vary by strain (p = 0.27 and p = 0.35). However, the proportion of MRSA infections that met the definition of nosocomial decreased significantly from 26.1% from 1999–2005 to 16.6% in 2006 (p = 0.0003). During 2006, patients infected with other MRSA strains were more likely than those infected with USA300/400 to have infections that met the definition of nosocomial (p = 0.0006).

**Table 1:** Descriptive epidemiology of invasive MRSA in Iowa, USA, 1999–2005*

Characteristic†	Total no. (%), N = 1,323	USA type		p value
		No. (%) USA300/400, n = 26	No. (%) other,‡ n = 1,297	
Mean age, y	67.8 (SD = 17.6)	46.0 (SD = 22.0)	68.2 (SD = 17.2)	<0.0001
Female gender	550 (41.6)	9 (34.6)	541 (42.7)	0.549
Inpatient stay	1,124 (85.0)	24 (92.3)	1,100 (84.8)	1.000
ICU admission	221 (16.7)	4 (15.4)	217 (16.7)	0.764
Nosocomial infection	346 (26.2)	5 (19.2)	341 (26.3)	0.306
Specimen type				<0.0001
Blood	1,256 (94.9)	25 (96.2)	1,231 (94.9)	
CSF	9 (0.7)	0	9 (0.7)	
Joint fluid	33 (2.5)	1 (3.9)	32 (2.5)	
Pleural fluid	8 (0.6)	0	8 (0.6)	
Other	6 (0.5)	0 (0.0)	6 (0.5)	
Iowa region				0.054
1	32 (2.4)	1 (3.9)	31 (2.4)	
2	370 (28.0)	10 (38.5)	360 (27.8)	
3	335 (25.3)	2 (7.7)	333 (25.7)	
4	272 (20.6)	4 (15.4)	268 (20.7)	
5	140 (10.6)	5 (19.2)	135 (10.4)	
6	63 (4.8)	4 (15.4)	59 (4.5)	
PVL	ND	ND	ND	ND
SCC*mec* IV	ND	ND	ND	ND

*MRSA, methicillin-resistant *Staphylococcus aureus*; ICU, intensive care unit; CSF, cerebrospinal fluid; PVL, Panton-Valentine leukocidin; SCC*mec* IV, staphylococcal chromosomal cassette *mec* type IV; ND, not done for all isolates. †The number of patients missing data on specific variables: age = 13; gender = 11; inpatient = 85; ICU = 356; nosocomial = 358; specimen type = 11; Iowa Department of Public Health Reporting Region = 11. ‡Of the subset of isolates that were typed (N = 180), 173 (96%) were USA100. The remainder clustered with USA200 (3), USA500 (2), or did not match an existing USA type.

**Table 2:** Descriptive epidemiology of invasive MRSA in Iowa, USA, 2006*

Characteristic†	Total no. (%), N = 343	USA type		p value
		No. (%) USA300/400, n = 54	No. (%) other,‡ n = 289	
Mean age, y	66.3 (SD = 17.0)	50.6 (SD = 21.2)	69.2 (SD = 14.4)	<0.0001
Female gender	135 (39.4)	14 (25.9)	121 (41.9)	0.059
Inpatient stay	278 (81.0)	50 (92.6)	228 (78.9)	0.271
ICU admission	56 (16.3)	8 (14.8)	48 (16.7)	0.348
Nosocomial infection	57 (16.6)	1 (1.9)	56 (19.4)	0.0006
Specimen type				0.0021
Blood	322 (93.9)	45 (83.3)	276 (95.0)	
CSF	0	0	0	
Joint fluid	13 (3.8)	5 (9.3)	8 (2.9)	
Pleural fluid	2 (0.6)	2 (3.7)	0	
Other	6 (1.7)	2 (3.7)	5 (1.4)	
Iowa region				0.268
1	10 (2.9)	0	10 (3.5)	
2	93 (27.0)	13 (24.1)	80 (27.7)	
3	49 (14.2)	12 (22.2)	37 (12.8)	
4	105 (30. 5)	16 (29.6)	88 (30.5)	
6	20 (5.8)	5 (9.3)	15 (5.2)	
PVL	54 (15.7)	52 (96.3)	2 (0.7)§	<0.0001
SCC*mec* IV	68 (19.8)	54 (100.0)	13 (4.5)	<0.0001

*MRSA, methicillin-resistant *Staphylococcus aureus*; ICU, intensive care unit; CSF, cerebrospinal fluid; PVL, Panton-Valentine leukocidin; SCC*mec* IV, Staphylococcal chromosomal cassette *mec* type IV. †The number of patients missing data on specific variables: age = 10; gender = 12; inpatient = 31; ICU = 122; nosocomial = 101; specimen type = 0; Iowa Department of Public Health Reporting Region = 6; PVL = 3; SCC*mec* IV = 3. ‡Of the subset of isolates that were typed (N = 272) 94% were USA100. The remainder clustered with USA200 (5), USA500 (5), USA600 (1), USA800 (4), or did not match an existing USA type. §Both isolates clustered with USA100 and were SCC*mec*II.

**Figure 1:**
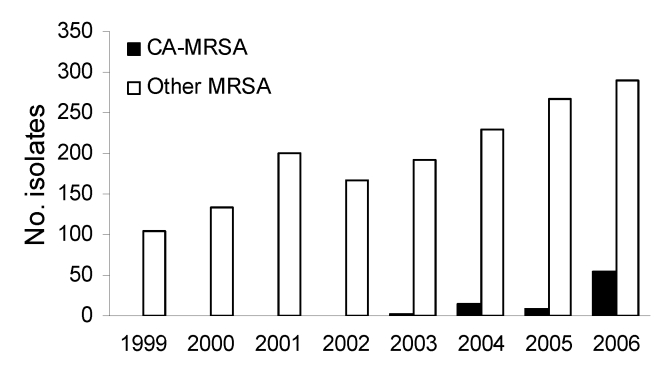
Number of invasive methicillin-resistant *Staphylococcus aureus* isolates submitted in Iowa, USA, 1999–2006. CA-MRSA, community-associated MRSA.

The antimicrobial susceptibility of 54 invasive USA300/400 isolates is shown in Table 3. None of the USA300 or USA400 isolates had a multidrug-resistant phenotype (e.g., all were resistant to <3 non–β-lactam classes). Specifically, none of the USA300 isolates from Iowa demonstrated the multidrug-resistance pattern described by Diep et al. that is mediated by the multidrug-resistance plasmid pUSA03 (*31*). All isolates were typeable when the DNA was digested with *Sma*I. We did not identify any invasive MRSA infections caused by USA300/400 between 1999 and 2002. USA300 caused 3 (1.5%) of 195 infections in 2003, 14 (5.8%) of 243 infections in 2004, 7 (2.5%) of 275 infections in 2005, and 51 (14.9%) of 343 infections in 2006. USA400 caused 2 (0.7%) of 275 infections in 2005 and 3 (0.9%) of 343 infections in 2006. The proportion of MRSA isolates from invasive infections that were CA-MRSA (either USA300 or USA400) increased significantly from 1999–2005 to 2006 (p<0.0001; Figure 1).

**Table 3:** Antimicrobial drug susceptibility of 54 invasive MRSA USA300/400 isolates, Iowa, USA, 2006*

Antimicrobial agent	% Susceptible
Erythromycin	9
Levofloxacin	57
Clindamycin†	93
Tetracycline	93
Mupirocin	98
Rifampin	98
Trimethoprim/sulfamethoxazole	100
Vancomycin	100
Gentamicin	100
Daptomycin	100
Linezolid	100

*MRSA, methicillin-resistant *Staphylococcus aureus*. †Includes D-testing of all erythromycin-resistant, clindamycin-susceptible isolates.

**Figure 2:**
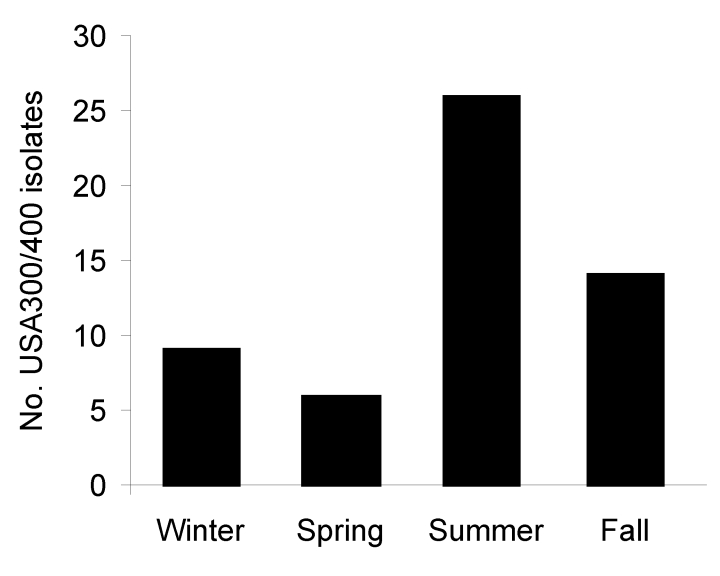
Number of USA300/400 methicillin-resistant *Staphylococcus aureus* (MRSA) isolates submitted by season, Iowa, USA, 2006.

Reporting region 4, which had the third largest population of the 10 regions, submitted the most isolates; region 1, which had the fourth smallest population, submitted the fewest isolates. We did not find significant differences between the type of MRSA causing infections and the reporting region during 2006. Incidence of infections caused by CA-MRSA varied by season during 2006 (p = 0.0004); a total of 47.3% of these infections occurred during the summer (Figure 2).

The full model for predicting invasive infection with CA-MRSA compared with HA-MRSA included age (young vs. old), seasonality, hospital exposure, and specimen type. However, the only significant predictors of CA-MRSA infection compared with HA-MRSA were age <69 years, which was associated with increased risk (odds ratio [OR] 5.1, 95% confidence interval [CI] 2.06–12.64), and hospital exposure (OR 0.07, 95% CI 0.01–0.51), which was associated with decreased risk.

## Discussion

The current study was unique because it evaluated invasive MRSA isolates from a statewide surveillance system in a rural area. Most prior studies of the epidemiology of CA-MRSA have focused on SSTI among patients in urban areas.

The published literature documents that incidence of CA-MRSA has increased over time in large urban areas. For example, Kaplan et al. found that incidence of CA-MRSA at Texas Children’s Hospital increased each year from August 1, 2001 to July 31, 2004 (*32*). The percentage caused by USA300 increased from ≈50% in 2000 to >90% in 2003. Of these infections, 95.6% were SSTI and 4.4% were invasive. EMERGEncy ID NET reported that USA300 caused 97% of the MRSA SSTIs seen in emergency rooms in 11 US metropolitan areas during August, 2004 (*15*). Seybold et al. demonstrated that by 2004 USA300 had become a common cause of MRSA healthcare-associated bloodstream infections (28%) and of nosocomial MRSA bloodstream infections (20%) at Grady Memorial Hospital in Atlanta (*21*). In contrast, the number of CA-MRSA (primarily USA300) isolates causing invasive infections did not increase substantially in Iowa until 2006.

Klevens et al. published a study of 8,987 invasive MRSA infections reported from the 9 sites in the Active Bacterial Core surveillance (ABCs/Emerging Infections Program Network) from July 2004 through December 2005 (*22*). The investigators conducted PFGE on 864 (11.3%) of the 7,648 isolates submitted from 8 sites. Of these isolates, 29% were USA300 (16% of the healthcare-associated, hospital-onset infections, 22% of the healthcare-associated, community-onset infections, and 67% of the community-associated, community-onset infections); <0.1% were USA400. In our study, 4.5% of all isolates we typed and 14.9% of isolates from 2006 were USA300, which suggested that the incidence of invasive infections caused by USA300 remains lower in Iowa than in the urban centers studied by Klevens et al.

Unlike the findings of Seybold et al. (*21*) and Klevens et al (*22*) from studies conducted in urban areas, USA300/400 rarely caused invasive nosocomial infections in Iowa, a rural state, during the study period. However, unpublished data from the University of Iowa Hospitals and Clinics indicate that these strains are becoming more common causes of invasive nosocomial infections, suggesting that the epidemiology of MRSA may be changing more slowly in rural areas than in large urban areas.

Diep et al. published a follow-up study of previous observations by Han et al. (*23*) about multidrug-resistant USA300 isolates (*31*). These investigators found multidrug-resistant isolates in Boston and in San Francisco and they identified male to male sex, past MRSA infection, and use of clindamycin to be risk factors for multiresistant USA300. A multidrug resistance plasmid (pUSA03) mediated these drug resistances. Fortunately, we did not identify this resistance phenotype among our USA300 isolates from Iowa. However, given the rapidity with which plasmid-mediated antimicrobial drug resistance can spread, and given the epidemic nature of USA300, we will continue surveillance for this and other antimicrobial resistances among USA300 isolates in Iowa.

Investigators in the Netherlands, Denmark, and Canada have found nontypeable MRSA among swine (*33**–**37*) and persons caring for swine (*33**–**36*). Strain ST398, which is not typeable by PFGE after DNA is digested with *Sma*I, has been found in all of these countries. Iowa produces ≈25% of the swine in the United States. However, we did not identify this strain among the invasive MRSA isolates submitted to the UHL.

Our data did not include information about preceding influenza infections, but we noted that CA-MRSA was isolated twice from the pleural space; other strains of MRSA were not isolated from this site. This finding suggests that CA-MRSA may have caused serious pulmonary infections in a few persons in Iowa. CA-MRSA, particularly USA300, has caused severe infections after influenza (or influenza-like) infections (*20**,**24*). During the influenza pandemic of 1918, Chickering and Park noted that many patients acquired severe secondary *S. aureus* pneumonias following influenza infections (*38*). Their observations suggest that coincident outbreaks of pandemic influenza and USA300 pneumonia could be catastrophic.

CA-MRSA, particularly USA300, is a public health concern for several other reasons. As noted previously, USA300 is rapidly replacing other strains of MRSA in the community (*15**,**31*) and has become an important nosocomial pathogen (*21*). Moreover, the types of infections caused by USA300 and the epidemiology of this strain are changing rapidly (*39*). Currently, most USA300 and USA400 isolates have fewer co-resistances than do HA-MRSA isolates (*13*). However, selective pressures can cause genetic drift in favor of more resistances; papers by Han et al. (*23*) and Diep et al. (*31*) documented that USA300 can acquire additional drug resistance determinants. If USA300 and USA400 become resistant to oral antimicrobial agents and the proportion of invasive MRSA infections caused by CA-MRSA continues to increase, many more patients will need parenteral vancomycin therapy, which will increase the difficulty and cost of treating these infections. Furthermore, as the incidence of CA-MRSA infections increases, horizontal transmission of these strains could increase in hospitals, making control of MRSA in healthcare settings even more difficult (*6**,**40*). Nosocomial bloodstream infections, ventilator-associated pneumonia, and surgical site infections caused by these strains could be devastating given the necrotizing nature of the infections.

Our study had several limitations. First, the surveillance system was passive. Consequently, demographic data and data about race/ethnicity and exposure to long-term-care facilities were incomplete and data about prior antimicrobial drug exposure and underlying diseases were not available. Additionally, we could identify the region where the specimen originated but not the specific city or county. Moreover, a validation study in Iowa found that hospital laboratories submit only 37% of the invasive MRSA isolates that they identify (D. Dufficy, pers. comm.). However, underreporting affected all regions equally. Furthermore, we used different selection criteria for typing invasive MRSA isolates submitted from 1999–2005 than we did for those submitted in 2006. We typed isolates from 1999–2005 only if they had <3 non–β-lactam coresistances, but we typed all invasive MRSA isolates from 2006. Thus, we may have introduced selection bias that would predispose the incidence of USA300/400 during 1999–2005 toward the null hypothesis (i.e., the annual proportion of MRSA isolates that were USA300/400 did not change during1999 to 2005) but away from the null hypothesis for the incidence of USA300/400 isolates during 1999–2006 (i.e., H_0_: The annual proportion of MRSA isolates that were USA300/400 was the same in 1999–2005 and in 2006). However, given that all the USA300/400 isolates identified during 2006 would have been detected using the 1999–2005 coresistance selection criterion, we believe that we typed all of the invasive USA300/400 isolates obtained during 1999–2005.

Some might argue that the incidence of invasive infections caused by CA-MRSA increased artificially because physicians were more aware of these organisms in 2006 than they were previously. CA-MRSA certainly has become a hot topic. CA-MRSA was initially identified in the mid 1990s, and many articles about these organisms have been published since then. However, the incidence of invasive infections caused by CA-MRSA in Iowa did not begin rising until 2006. Moreover, Iowa requires laboratories to send all invasive MRSA isolates to the UHL, and laboratory personnel are unlikely to know the details of each patient’s infection. Thus, laboratories probably would not submit isolates of 1 strain preferentially. Finally, many hospitals submitted isolates to the UHL, which suggests that submission bias was unlikely in 2006.

In conclusion, the number of invasive MRSA infections reported in Iowa and the number of invasive infections caused by CA-MRSA increased in Iowa from 1999–2005 to 2006. The increase of CA-MRSA (particularly USA300) poses a unique public health threat. Our study demonstrated that CA-MRSA no longer causes only SSTIs but now causes an increased proportion of invasive infections in a rural state. This finding is particularly disconcerting in light of the severity of these infections and the reports of necrotizing pneumonia caused by USA300 after influenza or influenza-like illness. Surveillance systems must continue to monitor the number and incidence of infections caused by USA300 and to monitor these isolates for changes in antimicrobial susceptibility. The relationship between seasons and CA-MRSA warrants further study.
